# A slanted castor wheel enables pushing manual wheelchairs from the side to improve social interaction

**DOI:** 10.1371/journal.pone.0307759

**Published:** 2024-09-12

**Authors:** Matto Leeuwis, Job Sesink, Lucy Bennett, Emma Schippers, Marieke W. Vassen, Bram T. Sterke, Katherine L. Poggensee, Heike Vallery

**Affiliations:** 1 Department of Biomechanical Engineering, Delft University of Technology, Delft, The Netherlands; 2 Department of Neuroscience, Erasmus University Medical Center, Rotterdam, The Netherlands; 3 Ziggy Mobility B.V., Delft, The Netherlands; 4 Department of Rehabilitation Medicine, Erasmus University Medical Center, Rotterdam, The Netherlands; 5 Institute of Automatic Control, RWTH Aachen University, Aachen, Germany; Instituto Tecnologico de Monterrey, MEXICO

## Abstract

Traditional wheelchairs are pushed from behind the occupant, which hinders eye contact and communication. It was proposed that the wheelchair be pushed from the side using a push bar to place the caregiver beside the occupant. However, this method requires the caregiver to exert continuous effort to maintain a straight trajectory because the force at the lateral push location generates a moment. This study explores simple modifications to the front castor wheel of the wheelchair that allow pushing it from the side without additional effort. We used a three-dimensional dynamic model of the castor wheel to predict the effects of altering its dimensions and rake, cant, and bank angle and present a simplified steady-state solution. Experimental results support the model’s predictions, and a proof-of-concept experiment with a wheelchair showed that it is possible to push a wheelchair from the side without increasing forces or moments. The results also confirmed that the lateral ground reaction force generated on the castor wheel is proportional to the normal force and the cant angle, which can counter the moment caused by the lateral push location. The implications of this model extend beyond wheelchair design and can be applied to other applications that use castor wheels, such as robotics, trolleys, and walkers.

## 1 Introduction

For some wheelchair users, such as children with Profound Intellectual and Multiple Disabilities (PIMD), face-to-face contact is crucial because they often rely on non-verbal communication [1]. Traditional wheelchairs place the caregiver directly behind the occupant, preventing eye contact and straining communication. A side-extending pushing bar was proposed to enable more natural interaction [2–6]. This placement allows the wheelchair user and caregiver to communicate as if they were walking beside each other. However, in contrast to the traditional wheelchair, where the net resistive and push forces act near the sagittal plane, a laterally extended push bar causes a free moment. As a result, the caregiver must deliver continuous effort to maintain a constant trajectory. Storch [7] proposed a clutch system that couples the rotation of the rear wheels to only allow straight-line movement, but this solution restricts mobility and is mechanically complex.

**Fig 1:**
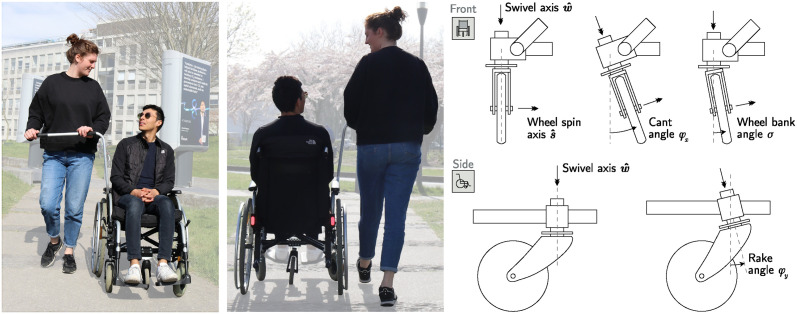
Definitions for the cant, rake, and wheel bank angles for a wheelchair castor wheel. *Left and middle:* A demonstration model of a wheelchair with a castor wheel with a cant angle. The castor wheel at the bottom of the wheelchair is visually slanted, allowing the caregiver to push from the side without applying a continuous moment to keep the wheelchair straight. An example of outdoor use is shown in S3 Video. The people depicted in the photos and videos gave written consent to publish as outlined in the PLOS consent form. *Top right:* The naming convention from ISO:7176:26 [8] is used to describe the castor wheel. A castor wheel is a wheel linked to a vehicle with a rigid connector, called the *castor fork*. The castor fork is connected to the vehicle with the castor stem and can rotate freely along the *swivel axis*, denoted here as $\hat{w}$. The rigid angle between the vehicle and the swivel axis $\hat{w}$ in the frontal plane defines cant angle *φ*_*x*_. *Bottom right:* The *rake angle* *φ*_*y*_ is the angle between the swivel and vertical axes in the sagittal plane. The *wheel bank angle σ* is defined as the angle between the swivel and wheel spin axes.

To alleviate the caregiver’s effort without restricting the movement of the wheelchair, here we propose a castor wheel with a non-vertical (i.e., slanted) swivel axis combined with a lateral pushing bar on the wheelchair. The swivel axis can be mounted in a non-vertical orientation by changing the rake or cant angle, as shown in Fig 1. This solution, in contrast to mechanisms that couple or lock the wheels [7], does not limit the mobility of the wheelchair as the swivel rotation is unconstrained and allows for straight and curved movements. While the general effectiveness of the cant or rake angle was demonstrated with a prototype [5], the relation between the design parameters and the generated lateral force exerted on the vehicle remains unknown. Preliminary results were reported in [5, 6].

Dynamical models for (castor) wheels with a rake angle have been developed for vehicles such as motorcycles, wave boards, and plane suspensions [9–12], but the cant angle is often ignored. For wheelchairs, a nonzero cant angle is generally considered a misalignment [8]. Garcia Agundez et al. [9] provide an elaborate dynamic analysis of the movement of a wave board with castor wheels, but the relation between lateral force and the design parameters cannot easily be inferred. A castor wheel with cant and rake angle was simulated by de Falco et al. [12], but only results for the effect of the rake angle are shown.

To push a wheelchair from the side, the castor wheel must generate a force or moment on the vehicle opposing the moment generated by the caregiver. The swivel axis cannot transmit an axial moment, so a lateral force could be applied to the vehicle instead. In this study, we intentionally mounted the castor wheel with a rake and cant angle to generate a lateral force on a wheelchair that facilitates pushing it from the side without additional effort. The goal was to predict the reaction forces on the modified castor wheel in steady state (curved trajectories with constant radius) using a three-dimensional dynamical model. Based on this model, a simplified equation was derived for quick manual calculations.

Simulations were validated using two experiments: The first measured an isolated castor wheel on a treadmill. The second compared a regular and a modified wheelchair being pulled at constant speed on a treadmill to demonstrate that the required push force does not increase when introducing a cant angle.

Overall, a cant angle could be used to push a wheelchair from the side without requiring additional pushing force as compared to a traditional wheelchair. These results can be generalized to simplify calculations and design castor wheels for various vehicles.

## 2 Materials and methods

We first parameterized and simulated regular and modified castor wheels to predict the generated lateral forces. The assumptions made in this work have been summarized in S1 Appendix. Next, we performed an experiment in which the forces and moments at the stem of the castor wheel were measured for different cant and rake angle combinations at various normal forces to validate prediction outcomes. In a second experiment, a regular and a modified wheelchair were pulled on a treadmill to demonstrate that the modified wheelchair could be pulled from the side without additional force.

### 2.1 Parameterization of the castor wheel

**Fig 2:**
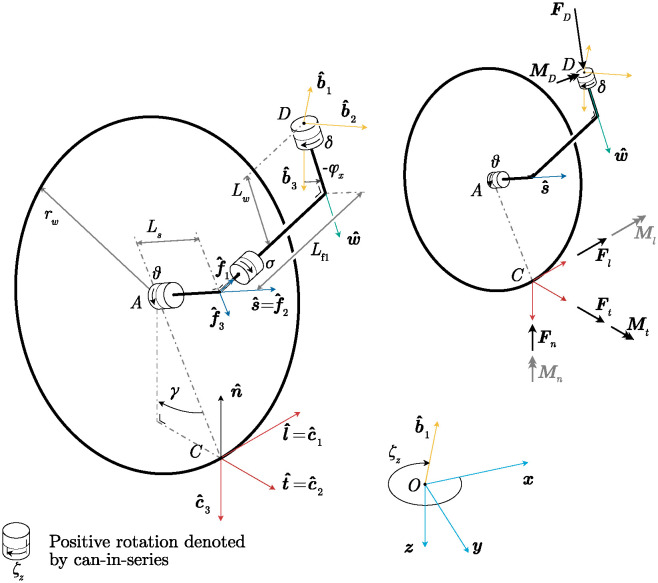
Kinematic and free body diagram of a modified castor wheel. *Left*: Kinematic diagram. Point *D* is the connection to the vehicle, point *A* is the connection between the castor fork and wheel, and *C* is the geometric contact point. The castor fork extends down from connection point *D* with length *L*_*w*_. The longitudinal castor trail *L*_*f*1_ is the minimal length of the castor fork between the swivel axis $\hat{w}$ and the wheel spin axis $\hat{s}$. A lateral trail *L*_*s*_ is defined along the wheel spin axis $\hat{s}$. Rotations are visualized using a cans-in-series [13] representation, where the positive rotation direction is indicated with an arrow. The wheel bank angle *σ* is illustrated using cans but is a solid connection. The rake angle *φ*_*y*_ is not shown for simplicity. A case equivalent to moving forward with a rake angle occurs if a castor wheel with a cant angle is moved in the ${\hat{b}}_{2}$ direction. *Right*: Free body diagram of the castor wheel. The force and moment on point *D* act on the vehicle, and the contact forces and moments at point *C* act on the ground. Moments that are assumed negligible in the model are colored gray.

This section defines the kinematics and parameters of the castor wheel. The full kinematic description can be found in S1 Appendix, where the kinematic locations of all important points are derived based on the design and state variables. The castor wheel has seven design variables: the cant angle *φ*_*x*_, the rake angle *φ*_*y*_, the wheel bank angle *σ*, the wheel diameter *r*_*w*_, the stem length *L*_*w*_, and the castor trail parameterized through *L*_*f*1_ and *L*_*s*_, all of which are shown and explained in Fig 2.

The castor wheel’s mass is considered negligible compared to that of the vehicle, so it is treated as an isolated, massless component. The castor fork is attached to a vehicle at point *D* and rotates with respect to the vehicle around the swivel axis $\hat{w}$. The swivel angle *δ* between the vehicle and the castor fork is defined as positive along $\hat{w}$. The castor wheel is modeled as a zero-thickness wheel with a radius *r*_*w*_ that rotates freely with respect to the castor fork along the wheel spin axis $\hat{s}$.

Orientations are defined using triads. A triad is a set of three orthonormal vectors that describe the orientation of one body with respect to one another. The inertial world coordinate system N (blue) is defined by the principal directions *x*, *y*, *z*, and the origin, point *O*. The triad B (yellow) is defined by the unit vectors ${\hat{b}}_{1}$, ${\hat{b}}_{2}$, ${\hat{b}}_{3}$, and is fixed to the vehicle. Triad F (dark blue) is fixed to the castor fork and is aligned with the wheel spin axis $\hat{s}$. Lastly, triad C (red) is the contact point triad and is aligned with the longitudinal contact direction $\hat{l}$ and normal vector of the ground plane $\hat{n}$.

The orientation of the swivel axis $\hat{w}$ with respect to the vehicle is defined with the cant angle *φ*_*x*_ and rake angle *φ*_*y*_. The wheel bank angle *σ* is the angle at which the wheel axis is mounted in the castor fork, parameterized using the angle between the wheel spin axis $\hat{s}$ and the swivel axis $\hat{w}$. The camber angle *γ* is the angle between the plane of the wheel and the ground plane and follows from the other design and state variables. These four angles are zero for traditional castor wheels on a horizontal surface.

Next, the generalized coordinates of the castor wheel can be described. The castor fork can freely swivel with respect to the vehicle by the swivel angle *δ*, and the wheel can spin with respect to the fork around the wheel spin axis with angle *θ*. Since the wheel is massless and has no rolling or bearing resistances, *θ* does not influence the dynamics. The location of point *D* is a function of the vehicle’s configuration. We assume that the vehicle prescribes the location of point *D* in the *x* and *y* direction, parameterized with *x*_*D*_ and *y*_*D*_, respectively.

Point *C* is always in contact with the ground plane, constraining the vehicle’s *z* location. The vehicle-fixed triad B always remains parallel to the ground plane and rotates with respect to the inertial triad N by a yaw angle *ζ*_*z*_. In summary, the “vector” ***q*** containing generalized coordinates of the castor wheel is

$$\begin{array}{c} q={\left(\begin{array}{ccccccccc} \delta & & \theta & & \zeta_{z} & & x_{D} & & y_{D} \end{array}\right)}^{T}. \end{array}$$ (1)

Locations of points are defined using relative position vectors in the form of rFA/D, which describes the location of point *A* with respect to point *D* when expressed in the triad F. The position of the center of the wheel *A* with respect to the vehicle connection point *D* follows from the geometry and is a function of the swivel angle *δ*. In the fork-fixed triad F, the relative position vector is

rFA/D=(-Lf1-Lw-LssinσLscosσ). (2)

The position of the contact point *C* with respect to point *A* can be defined by finding a vector in the wheel plane that is perpendicular to both the wheel spin axis $\hat{s}$ and the longitudinal contact direction of the wheel $\hat{l}$. Using the wheel radius *r*_*w*_, the position vector follows from the cross product

$$\begin{array}{c} r_{C/A}=r_{w}\cdot \hat{l}\times \hat{s}. \end{array}$$ (3)

Together, Eqs 2 and 3 define the location of the contact point with respect to the vehicle connection as a function of the coordinate vector ***q***. The full derivation of the kinematics, including how directions $\hat{l}$, $\hat{s}$ and $\hat{t}$ are computed, can be found in S1 Appendix.

### 2.2 Modeling reaction forces

No inertial terms are present since the castor fork and wheel mass are ignored. Therefore, static equilibrium holds for this component, and all loads can be derived from only the sum of forces and moments around point *D*. Forces on the castor wheel are defined in the free body diagram in Fig 2. The net force at the contact point *C* is equal to

$$\begin{array}{c} F_{C}=F_{t}+F_{n}+F_{l}\;, \end{array}$$ (4)

where

***F***_*t*_ is the lateral tire force, with known direction $\hat{t}$ and unknown magnitude *F*_*t*_;

***F***_*n*_ is the normal force, with known direction $\hat{n}$ and unknown magnitude *F*_*n*_;

***F***_*l*_ is the longitudinal tire force, with known direction $\hat{l}$ and unknown magnitude *F*_*l*_.

The net sum of forces can then be calculated using

$$\begin{array}{c} F_{D}+F_{C}=0, \end{array}$$ (5)

and the sum of moments around point *D* is

$$\begin{array}{c} M_{D}+M_{t}+r_{C/D}\times F_{C}=0, \end{array}$$ (6)

where

***F***_*D*_ is the force from the vehicle on the castor fork at point *D*, with three unknown components;

***M***_*D*_ is the moment on the castor fork at point *D*, with three unknown components;

***M***_*t*_ is the rolling resistance moment, with known direction $\hat{t}$ and unknown magnitude *M*_*t*_;

***r***_*C/D*_ is the position vector of point *C* with respect to *D*, which is assumed to be known from the kinematics (see S1 Appendix);

**0** is a vector of zeros.

Eqs 5 and 6 consist of six scalar equations, one for each force and moment component. However, they also contain ten unknowns: the three elements of the vehicle reaction force ***F***_*D*_ and moment ***M***_*D*_, the three elements of the contact force ***F***_*C*_, and the rolling resistance moment *M*_*t*_. Therefore, four additional constraints or assumptions are required to solve the system.

If we assume that the vehicle drives in a steady state, the magnitude of the normal force *F*_*n*_ is constant and determined by the vehicle’s loading (i.e. the weight distribution across the wheels, roughly a third of the weight on the front wheels for wheelchairs [14]). The assumption of steady state was made to separate the dynamics of the vehicle from the general equations derived here. Due to the inertia of the vehicle, the normal force would be a function of the rate of change of the swivel angle as it causes a vertical displacement. We defined this steady state such that the castor wheel rolls at constant velocity and the first and second time derivatives of the swivel angle *δ* are zero. For a wheelchair, this corresponds to a case where the wheelchair is propelled at a constant forward and angular velocity by a caregiver who applies both a pushing force and a free moment. Design parameters can then be chosen so that the free moment is small or zero when the wheelchair is pushed from a lateral location.

The rolling resistance is modelled using a longitudinal tire force *F*_*l*_ acting against the direction of movement of the wheel and a rolling resistance moment *M*_*t*_. The magnitude was calculated using a fixed rolling resistance coefficient *f*_*r*_ [15]. We assume the rolling resistance coefficient does not depend on the camber angle *γ*, and the camber angle is small, such that the vertical distance between contact point *C* and the wheel center *A* is always equal to the wheel radius *r*_*w*_. Since there was assumed to be no friction in the wheel spin bearing, the rolling resistance moment must be opposite to the moment generated by the longitudinal tire force. The rolling resistance moment was assumed to lie in the ground plane along $\hat{t}$. The longitudinal force and rolling resistance moment, assuming that the castor wheel is moving in the $\hat{l}$ direction, are then equal to

$$\begin{array}{c} F_{l}=-F_{n}\;f_{r}, \end{array}$$ (7)

$$\begin{array}{c} M_{t}=-F_{l}\;r_{w}. \end{array}$$ (8)

Lastly, if we assume that there is no friction in the swivel axis, the component of the moment on the castor fork ***M***_*D*_ in the direction of the swivel axis $\hat{w}$ must be zero:

$$\begin{array}{c} M_{D}\cdot \hat{w}=0. \end{array}$$ (9)

With these four additional constraints, the reaction forces ***F***_*D*_ and moments ***M***_*D*_ on the vehicle can be uniquely determined if the generalized coordinates $\hat{q}$ (Eq 1) are known. Since the wheel spin angle *θ* does not influence the dynamics, and *x*_*D*_, *y*_*D*_, and *ζ*_*z*_ parameterize the location and orientation in space, knowing only *δ* is sufficient to solve the reaction forces in steady state. If transient dynamics are of interest as well, this model could be extended by finding the equations of motion of the whole vehicle, which was done for a wheelchair by Leeuwis [6]. In transient conditions, the normal force *F*_*n*_ is a function of the vehicle’s inertia (e.g. due to height changes caused by a changing swivel angle or load transfer due to acceleration).

### 2.3 Simplified equation for a cant-only castor wheel

The equilibrium equations can be simplified with additional design parameter assumptions. Imagine a traditional castor wheel where only a small cant angle *φ*_*x*_ is introduced. In this case, the lateral castor trail *L*_*s*_, rake angle *φ*_*y*_, and wheel bank angle *σ* are zero, and the cant angle can be simplified using a small-angle approximation. For this configuration the points *A*, *C*, and *D* coincide with the plane spanned by $\hat{w}$ and $\hat{l}$. Therefore, the longitudinal tire force ***F***_*l*_ does not cause a moment around the swivel axis $\hat{w}$. The moment *M*_*t*_ has a small contribution as $\hat{t}$ is not perpendicular to $\hat{w}$; this contribution is ignored in the simplified solution derived here.

If we use the virtual work method on a steady-state trajectory, only the lateral tire force *F*_*t*_ and force on the castor stem *F*_*D*_ have displacements as a function of *δ* capable of doing virtual work. The change in height due to an infinitesimal change of swivel angle *∂δ* is equal to

$$\begin{array}{c} \partial r_{n}=L_{f1}\varphi_{x}\;\text{cos}\;\left(\delta \right)\;\partial \delta , \end{array}$$ (10)

and the lateral displacement of point *C* in direction $\hat{t}$ is

$$\begin{array}{c} \partial r_{t}=-L_{f1}\;\partial \delta . \end{array}$$ (11)

The magnitude of the lateral force can be approximated with the fact that virtual work *∂W* in static equilibrium is zero

$$\begin{array}{c} \partial W=\partial r_{t}\cdot F_{t}+\partial r_{n}\cdot F_{n}=0, \end{array}$$ (12)

resulting in the simple equation

$$\begin{array}{c} F_{t}=\varphi_{x}F_{n}\;\text{cos}\;\left(\delta \right). \end{array}$$ (13)

This approximation is only valid for small cant angles. It ignores roll resistance and nonlinear changes of contact point location due to swivel angle, but it does provide a simple, easy-to-implement equation that can be used over the full range of swivel of the castor wheel. Furthermore, this solution is also valid for any combination of small rake and cant angles, as the rake angle can be parameterized indirectly through the cant and swivel angle. For example, a castor wheel with a positive rake angle is equivalent to one with a positive cant angle and a swivel angle of −90 degrees.

It is also assumed that, since the cant angle is small, the swivel angle *δ* is equal to the angle between the longitudinal contact vector $\hat{l}$ and the forward direction of the vehicle ${\hat{b}}_{1}$. This assumption is always valid in a traditional castor wheel. However, if the swivel axis is non-vertical, a nonlinear relation exists between the wheel radius and the location of contact point *C*. This nonlinear effect is magnified for castor wheels that have a large wheel radius compared to the castor trial. In those cases, the complete equations described in Eqs 5 and 6 must be used, which can account for kinematic nonlinearities and the effect of roll resistance in more complex configurations.

### 2.4 Design of an experimental setup to validate the castor wheel model

**Fig 3:**
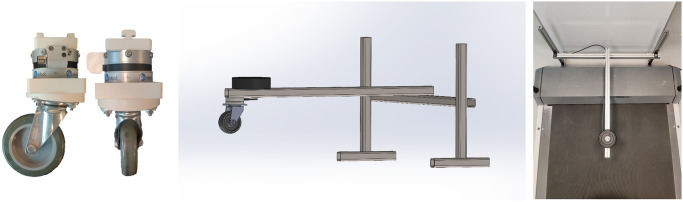
Overview of the instrumented castor wheel on the treadmill. *Left:* side and front view of the castor wheel with a wedge-shaped connector and force sensor. The round white wedge defining the rake and cant angle is mounted directly to the castor plate. The wedge was rotated with respect to the force sensor to vary the swivel angle. *Middle:* CAD drawing of the castor wheel set-up. The beam can freely rotate at the H-frame, and weights (black) were used to increase the normal force on the castor wheel. *Right:* Top view of the castor wheel set-up on the treadmill. The castor wheel is located directly under the black weight.

An experiment with a castor wheel on a treadmill was used to validate the simplified lateral force prediction in Eq 13. The castor wheel was placed on a treadmill moving at a constant speed with a three-bar assembly, and a variable amount of weight was placed on top as shown in Fig 3. An OnRobot HEX-H-QC six-axis torque and force sensor was used to measure the forces and moments at point *D*. The castor wheel had a radius *r*_*w*_ of 9.5 cm and a (longitudinal) trail *L*_*f*1_ of 5 cm.

Three different experimental sets were performed, and during each, only one variable was varied: (1) cant angle *φ*_*x*_, (2) normal force *F*_*n*_, and (3) swivel angle *δ*. The first two sets validate the linear dependence of the lateral tire force *F*_*t*_ on the cant angle and normal force, and the last set validates whether changing the swivel angle results in a sinusoidal lateral force as predicted in Eq 13.

Normal force was changed by varying the weight placed on the beam, resulting in loads of 19.8 N, 39.3 N, 57,5 N, and 75.5 N. The swivel angle was varied by rotating a round white wedge with respect to the direction of movement in increments of 45°. Multiple wedges were used to test cant angles of 0, 0.05, 0.1, 0.15, and 0.2 radians. It was assumed that, due to the small cant angles, the swivel angle was equal to the angle change in the direction of movement. A change in swivel angle can represent multiple steering angles of a castor wheel with a cant angle, rake angle, or a combination of both. For example, a swivel angle of 90 degrees on a canted castor wheel is equivalent to a swivel angle of 0 degrees on a castor wheel with only a rake angle.

### 2.5 Proof of concept with a wheelchair on treadmill

**Fig 4:**
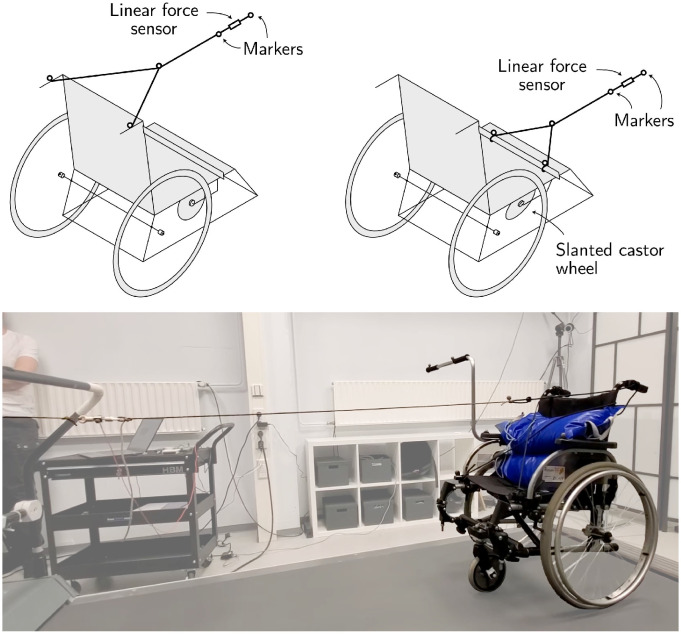
Overview of the proof-of-concept wheelchair on the treadmill. *Top left:* Control condition. The wheelchair was pulled forward by a rope attached to the rear handles in the original configuration. A linear force sensor measured the tension in the rope, and the direction of that force vector was determined by markers placed along the rope and handles. *Top right:* Test condition. The wheelchair was pulled forward by a rope attached to the push bar in the configuration, which includes a wheel with a cant angle of 0.1 radians. *Bottom:* Experimental setup. The wheelchair was pulled forward on a treadmill, carrying a 40 kg weight to simulate a human sitting in the device.

A proof-of-concept measurement with a three-wheeled wheelchair on a treadmill was used to evaluate whether a castor wheel with a cant angle allows pushing a wheelchair from a lateral location. A three-wheeled design was specifically chosen because the modification could be implemented as an additional castor wheel that lifts the two existing castors. By using an add-on, the slanted castor wheel could be attached and removed depending on the needs of the current situation, though this prototype was not designed to streamline this process (see Discussion). The modular wheelchair carrying a 40 kg weight was rolling along a level treadmill (setup shown in Fig 4, S1 and S2 Videos), and a measurement was performed in the control condition and a test condition. Both conditions were achieved using an additional castor wheel temporarily attached to the wheelchair in between the attachment point of the regular castor wheels. A wedge-shaped connector was used to achieve a cant angle of 0.1 radians in the test condition.

The wheelchair was pulled forward at a speed of 3 km/h, using a rope held by the same experimenter in both conditions. The pulling force (equivalent in magnitude to the pushing force) was measured by a linear force sensor (FUTEK LSB200, 5 lb, JR S-Beam load cell). In the control condition, the rope was attached to both rear handles on a standard wheelchair, and the swivel axis was in a vertical orientation (no cant or rake angle). In the test condition, a wedge-shaped connector was used to set the castor wheel at a cant angle of 0.1 rad, and the rope was attached to the laterally extending handlebar to simulate pushing from the right side. For both conditions, the wheelchair was initially stabilized by an experimenter until it reached a steady-state forward speed, which was then maintained and measured for at least ninety seconds. A short video recording of both conditions is included in S1 and S2 Videos.

An optical motion capture system (Qualisys AB, Sweden) measured markers placed along the pulling rope and at the wheelchair handles where the force was applied to determine the angle of the pulling force relative to the wheelchair’s movement. Because the rope was not constrained to the forward direction, the pulling force was calculated as the projection on the direction of movement of the wheelchair. The angle of the rope was defined by the marker at the intersection of the ropes attached to the handle and the midpoint between the two markers on either side of the force sensor. A *t*-test was then performed to test the significance of the forces required to pull the wheelchair in each condition for *α* = 0.05; we assumed normality for the analog noise in the force sensor and tracking error of the motion capture system. The validity of the test was also examined by measuring the normal force on the castor wheel in each condition using a standard kitchen scale; a *t*-test was also performed to determine significance.

## 3 Results

First, the results from the experiments with the castor wheel on the treadmill are used to compute rolling resistance and compared to simulated predictions. Second, the feasibility of pushing a wheelchair with a canted castor wheel from the side is assessed from the experimental proof of concept. A demonstration video of the prototype is provided in the S3 Video, and data and processing code are provided in the S1 File.

### 3.1 Model validation with experimental data

First, the rolling resistance coefficient of the castor wheel on the treadmill was computed by dividing the average force in the forward direction by the normal force in the trials that used the maximal weight. The rolling resistance coefficients for the cant angles *φ*_*x*_ of 0, 0.05. 0.1, 0.15, and 0.2 radians were equal to 0.025 ± 0.014, 0.019 ± 0.017, 0.026 ± 0.016, 0.024 ± 0.017, and 0.031 ± 0.016, respectively. A linear regression was conducted to examine the relationship between cant angle and rolling resistance. The cant angle coefficient was not significant (*β* = 0.033, p = 0.232), indicating that the cant angle had no significant effect on the rolling resistance. The mean rolling resistance factor of 0.025 is used to calculate rolling resistance following Eq 7.

**Fig 5:**
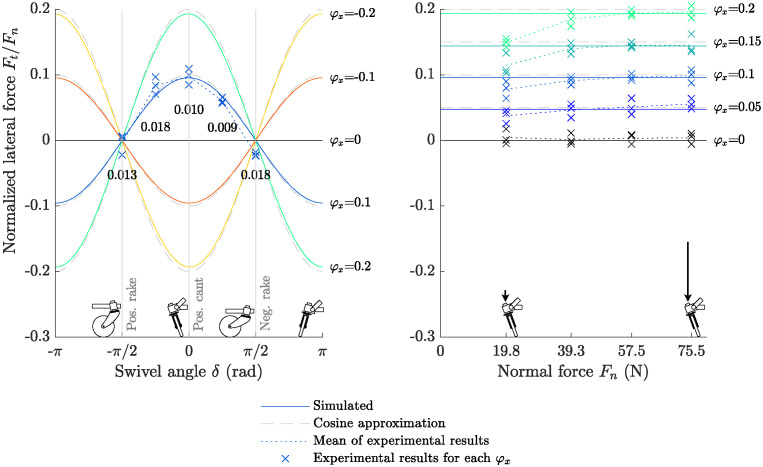
Lateral force on castor wheel as measured with the instrumented castor wheel on the treadmill, compared to simulated predictions. *Left:* Normalized lateral tire force *F*_*t*_ as a function of the swivel angle *δ* for multiple cant angles *φ*_*x*_. Solid lines correspond to the full system given in Eqs 5 and 6; dashed lines correspond to Eq 13. Experimental results for a cant angle of *φ*_*x*_ = 0.1 *rad* (blue crosses for data points, dotted line for mean) were collected. The RMSE over the 3 trials with respect to the simulation is printed below the crosses. The pictographs of the front and side view of the castor wheel on the swivel angle axis depict a castor wheel with a positive cant angle under the given swivel angles. A rake angle (or combination of cant and rake) can be read from the graph at *δ* = −90°, as a rake angle is equivalent to a cant angle when the direction of movement is rotated by 90°. *Right:* Normalized lateral tire force *F*_*t*_ as a function of the normal force *F*_*n*_ for multiple cant angles *φ*_*x*_. Solid lines correspond to the simulated system. The normal force is read from the force at the force sensor and does not include the weight of the castor wheel and fork. Experimental results (blue crosses for data points, dotted line for mean) were collected for each shown cant angle.

The Eqs 5 and 6 (‘Simulated’) included rolling resistance and the system’s full kinematics as described in S1 Appendix. The simplified Eq 13 (‘Cosine approximation’) predicted that the lateral force *F*_*t*_ on the castor wheel was proportional to the cant angle *φ*_*x*_ and normal force *F*_*n*_, and followed a cosine dependence on the swivel angle *δ*. Fig 5 depicts the normalized lateral force on the castor wheel as predicted with the simulated system (solid lines) and the cosine approximation (gray dashed lines) for various cant angles (left) and normal forces (right). The largest observed error between both predictions was 0.0045 and 0.0089 normalized lateral force for *φ*_*x*_ as 0.1 and 0.2 radians, respectively. The root mean square errors of the force were 0.0033 and 0.0061, respectively, suggesting that both solutions are comparable. When the largest rolling resistance value estimated by Sauret et al. for a castor wheel radius of 9.5 cm [15] (0.035, standard wheel on carpet) was used, the maximal observed difference of normalized lateral force increased to 0.0065 and 0.0129 for *φ*_*x*_ as 0.1 and 0.2 radians, respectively. Without rolling resistance, the maximal error was only 0.0003 and 0.0027. Overall, both solutions resemble each other closely, suggesting that the cosine approximation may be a sufficient description of the system unless the rolling resistance is large.

Next, the simulation was compared to the three experimental series that varied (1) cant angle *φ*_*x*_, (2) normal force *F*_*n*_, and (3) swivel angle *δ*. The results of these three experimental series are shown in Fig 5 (crosses for trial means, dotted line for the mean of all trials, RMSE between data and simulation printed). The swivel angle *δ* was only varied at a cant angle of 0.1 rad and a normal force of 60.3 N (Fig 5, left). The normal force and cant angle were varied in a grid for the case where the swivel angle was zero (Fig 5, right). The normal force was approximated using the measured vertical force on the sensor. This weight excludes the mass of the castor wheel and fork, which were assumed to be massless in the simulations.

The experimental results where cant and rake were varied matched the simulated system well (Fig 5, left; max. RMSE 0.022). The simulation and experimental results deviated more when the normal force was low, reaching on average 0.178 when *φ*_*x*_ = 0.2 rad and *F*_*n*_ = 19.8 N (Fig 5, right). The mean error was considerably smaller at higher normal forces; at 75.5 N, the largest mean error was 0.0079 normalized lateral force. Overall, experimental data matched the simulation when the normal force was sufficiently large.

### 3.2 Wheelchair experiment

The proof-of-concept measurement with the wheelchair on the treadmill demonstrated the feasibility of maintaining a steady-state trajectory using a pulling force. Videos of both experimental conditions are provided as supplementary material (S1 and S2 Videos). In both conditions, the wheelchair rolled steadily on the treadmill without major deviations for at least ninety seconds. The normal force at the castor wheel was 48.9 N in both conditions, with no significant difference (*t*-test, *t* = 0.13, *p* = 0.90). The force required to maintain steady-state forward motion was 4.55 N in the control condition and 4.56 N in the canted condition. These pulling forces were statistically similar (*t*-test, *t* = 0.01, *p* = 0.99), suggesting that the introduction of the cant angle did not affect the force required to propel the wheelchair.

## 4 Discussion

An increase in the cant angle leads to a proportional increase in lateral force generation as a function of the swivel angle (Fig 5). This makes it a suitable modification to generate a lateral force on a vehicle, which can compensate for the free moment generated by the caregiver pushing from a lateral location. In the proof-of-concept experiment with the wheelchair, it was demonstrated that no free moment is required to push the wheelchair from a lateral position on the handlebar. The system presented in Eqs 5 and 6 and the cosine approximation of Eq 13 were found to be similar for the simplified castor wheel. Therefore, we accept both predictions and recommend the simplified Eq 13 to estimate lateral forces for castor wheels that meet the equation’s assumptions (see S1 Appendix). The S3 Video provides an outdoor demonstration video where the modification is used to traverse straight and curved paths.

### 4.1 Push force is not increased by cant angle

Resistive forces (e.g. rolling resistance and bearing friction) were assumed to contribute little to the lateral force in Eq 13. This simplified equation, omitting numerous effects such as bearing friction and nonlinear kinematics, accurately predicted the force on the castor wheel in Experiment 1, indicating that this simplification can be justified for this special case. Furthermore, both experiments demonstrated that the resistive force did not increase when introducing the cant angle. This implies that the caregiver can push a traditional and modified wheelchair with a similar effort in a straight trajectory. However, we caution that the rolling resistance can still affect the caregiver’s applied forces and moments depending on the vehicle’s design. For example, if the front wheel is not placed on the sagittal plane, the roll resistance generates a vertical moment vector, affecting the vehicle’s rotation in the yaw direction.

One explanation for the unchanged resistance forces is that the lateral tire force, by its used definition, is tangent to the direction of movement and cannot do work for a thin wheel without slip. In reality, a small lateral slip is present and could increase with large lateral forces. Then, a lateral force can do work and increase rolling resistance. Since the proposed solution in Eq 13 was a good fit for the data, the lateral slip was not included in this model. A more detailed account of kinematics and the influences of tire mechanics is provided in S1 Appendix. It was previously estimated that a wheelchair could be pushed from the side with a cant angle of 0.05 radians [6]. The corresponding normalized lateral force is 0.05, orders of magnitude smaller than the friction coefficients of rubber wheels. Therefore, it is unlikely that a wheel’s static friction coefficient is insufficient to prevent slip when generating this lateral force.

The lateral force on the castor wheel generated by the cant angle scaled linearly with the normal force on the wheel. Similarly, the rolling resistance of wheelchairs is mostly proportional to the normal force [15–17]. Because the rolling resistance and lateral tire force both scale proportionally to the normal force on the wheel, the required cant angle to maintain a constant trajectory does not vary with the weight of the wheelchair occupant. This is, however, only the case under the assumption that the weight distribution between the front and rear wheels remains unchanged.

### 4.2 Validity

The exact calculation and cosine fit matched closely when only the cant angle is varied (Fig 5), quantified by low RMSE values when the normal force was at 57.5 N or higher. This relation was demonstrated to be valid for cant angles from 0 to 0.2 radians (11.46 degrees). However, the error between the simulation and experiments was larger at low normal forces. Since normal force was measured at the castor stem, the weight of the castor fork and wheel were not included. Furthermore, the assumption that the castor wheel and fork are massless compared to the load they carry might not be valid if the load is small.

For regular wheelchairs, it is assumed that a third of the total weight is on the castor wheels [14]. For a total (occupant and wheelchair) weight of 60 kg, this would result in a normal force of 198 N, whereas the simulations and results form a good match from 57.5 N and higher. It was impossible to perform experiments with higher weights in the current experimental setup due to the maximal safe moment of the six-axis force sensor.

The experiments did not vary the wheel bank angle or lateral castor trail, as the cant and rake angle achieved the desired effect of generating controlled lateral forces. The system presented in Eqs 5 and 6 does allow for predictions where these parameters are changed, but no validation data was collected here. In earlier work, we predicted that wheel bank angle might have utility to fine-tune the shape of the lateral force curve [6], but doing so might result in increased lateral slip of the wheel (see S1 Appendix). The model predicts that the castor trail minimally affects the magnitude of the lateral tire force. This prediction relies on the fact that both the moment arm of the lateral tire force and the normal force scale with castor trail. The contact point must have a sufficient trail behind the intersection of the swivel axis with the ground plane. Otherwise, the system may become unstable due to a phenomenon called castor shimmy [10]. Furthermore, the influence of nonlinear kinematics is amplified if the castor trail is small compared to the castor wheel radius.

### 4.3 Practical considerations

A slanted castor wheel allows the caregiver to push the wheelchair from the side. However, some practical limitations should be considered.

First, a canted castor wheel causes swivel angle-induced height changes. The changes in height as a function of the swivel angle are minor; for the currently used castor wheel, the maximum vertical change between the lowest and highest point was equal to 10 mm. Yet, a stiff wheelchair frame could cause the normal force on two canted castor wheels to depend on their swivel angle, complicating the accurate modelling of the vehicle. An early functional prototype used one traditional and one slanted castor wheel. Here, we opted to use a three-wheeled wheelchair instead. If the castor wheel can move freely, its energetic preferred position is where the mass (point *D*) is lowest. For the simple castor wheel, this corresponds to aligning the longitudinal direction $\hat{l}$ against the projection of the swivel axis $\hat{w}$ on the ground plane [6]. The rake and wheel bank angle could be used to modify the neutral position of the wheel.

Second, this height change causes the castor wheel to orient itself to a preferred orientation when no other forces are applied. When starting a movement, the wheel reorients itself to the direction of movement, but this requires additional work. This problem would be particularly evident in daily use, as the median wheelchair bout distance is only 8 m [18]. Furthermore, walking beside an occupant for continuous indoor use would not be feasible, similar to how walking side by side continuously is infeasible during short bouts in healthy gait [19].

Third, the caregiver must apply a free moment to maintain a constant trajectory when driving uphill or downhill. The push force is increased or decreased due to the longitudinal component of gravity, while the normal (and therefore lateral) force on the castor wheel does not change considerably. As a result, the yaw moment is not in equilibrium. This work assumed that the wheelchair rolls on a flat and horizontal surface. Manual breaks, similar to the ones on bicycles, could be used to slow or stop the wheelchair while pushing from the side in the case of downhill driving [2]. In the case of uphill driving, the increased push force of the caregiver generates a moment that the caregiver must also compensate by either pushing closer to the saggital plane or applying a free moment.

To address the above problems, we propose that the cant angle and lateral extending push bar should operate as an add-on or toggle only for longer bouts. The current prototype’s lateral-extending push bar could be folded quickly, but the castor wheel with the cant angle remained attached to the wheelchair. Other wheelchair modifications, such as the Freewheel [20], add a single front wheel as a temporary modification. The currently used slanted castor wheel similarly operates as an add-on that could transform a wheelchair from four to three contact points, where the normal castor wheels remain attached to the frame. Future designs could employ a toggle mechanism, perhaps coupled to the folding of the lateral extending push bar, to change the cant angle during daily use. A toggle-able cant angle could even aid traditional wheelchair use (pushed or manually propelled) while driving on a banked incline.

### 4.4 Structural considerations

It was demonstrated that the modified castor wheel can generate the required forces to allow a caregiver to push the wheelchair from a lateral location. However, this modification may also increase the mechanical load on the components. We identified the main points of failure as (1) the bending moment on the swivel bearing, (2) the bending moment and axial load on the wheel bearing, (3) the structural strength of the vehicle, and (4) the durability of the tire.

The swivel bearing’s bending moment increases due to the introduction of the lateral force *F*_*t*_. In a normal castor wheel, most of the bending moment is caused by the normal force acting with a moment arm on the bearing due to the castor trail. For the castor wheel simulated in Fig 5, the maximal normalized bending moment on the swivel axis was equal to 0.054, 0.071, and 0.088 Nm per N of the normal force for cant angles of 0, 0.1, and 0.2 radians. This is an increase of 31% and 63% compared to a traditional castor wheel, respectively. We acknowledge and caution that increased bending moments could reduce the longevity of the bearing and could reduce the maximal weight that can safely be carried.

Second, we consider the bending moment and radial load on the castor wheel’s wheel bearing. A traditional castor wheel swivels freely, meaning that the axial load on the wheel spin bearing is generally small. For a castor wheel with a cant angle, a bending moment consists of components generated by *F*_*n*_ and *F*_*t*_. A phenomenon called camber thrust causes the net reaction force to generally fall near the plane of a (castor) wheel during movement [21–24]. As a result, the axial load is not severely increased in castor wheels operating at a nonzero camber angle. This is further discussed in S1 Appendix. In a static position, there is an axial load equal to the sine of the camber angle times the normal force. A regular castor wheel ball bearing proved sufficient for our proof-of-concept, though the authors recommend selecting a bearing rated to withstand the radial load, axial load, and bending moments of the intended application.

Third, the vehicle’s resilience to withstand a continuous lateral force and lateral push location could be questioned. Wheelchairs specifically are subjected to tilt testing, in which a wheelchair is loaded and placed on an incline that tilts the wheelchair in either the roll or pitch direction [25]. Doing so tests the frame’s strength in the longitudinal and lateral direction, confirming that the vehicle can withstand substantial lateral loads on the castor wheel connection point. The lateral extending location of the push bar also introduces a large vertical moment on the wheelchair frame. The current location, attachment method, and design of the push bar were not intended to be final and were not the focus of this study. Future work must consider specific wheelchair designs and evaluate whether the structural integrity of existing frames is sufficient.

Lastly, the structural strength of the tire itself should be considered. Many wheels, such as the ones on bicycles [23, 24, 26], operate under a camber angle temporarily, whereas the rear wheels of some wheelchairs are placed under a large camber angle permanently [27]. The scope of this study was limited to investigating the feasibility of using a castor wheel with a cant angle to push a wheelchair from the side and did not include extensive wear testing. No failures or notable wear occurred during a 6-week pilot where modified wheelchairs with a cant angle were used for normal daily activities. Future work could investigate what bearings and wheels can withstand long-lasting use under a cant angle.

### 4.5 Conclusion

In summary, the cant angle proved a suitable design parameter to increase the lateral force applied by a castor wheel on a vehicle, which allowed the caregiver to push the wheelchair using the push bar without additional effort. Eq 13 provides a simple-to-use method to calculate lateral forces for simple castor wheels and shows the linear dependence of lateral force on normal force and small cant angles. The castor trail was predicted not to influence lateral force generation but should be sufficiently large to prevent instability. The rake angle modifies in which direction of vehicle movement the lateral force is maximal, which can be tuned depending on task and vehicle requirements. This modification can be applied to any vehicle that could benefit from asymmetric propulsion, such as wheelchairs, carts, robotic systems, and mobility aids. Future work could also investigate dynamic stability under higher velocities and alternative solutions to facilitate pushing a wheelchair side by side without increased effort.

## Supporting information

S1 Appendix Description of kinematics and tire forces. (PDF)

S1 File Experimental data and the MATLAB code for calculations of castor wheel. Contains the data collected during the validation experiments and the code for calculating the reaction forces for a castor wheel using the vector equations and the cosine approximation. 4TU.ResearchData repository: https://doi.org/10.4121/82afa4e1-bdf1-4bf4-8682-32e62f6c3d68, GitLab repository: https://gitlab.tudelft.nl/ziggy/evaluation_paper.
(ZIP)

S1 Video Video of the control wheelchair on the treadmill. The video contains the control conditions, where the wheelchair was pulled using a cable while rolling on a treadmill.
(MP4)

S2 Video Video of the test wheelchair on the treadmill. The video contains the test condition, where the wheelchair with a slanted castor wheel was pulled using a cable attached to the push bar while rolling on a treadmill.
(MP4)

S3 Video Video of the wheelchair during outdoor use with an occupant. The video demonstrates how a modified wheelchair could be used outdoors on an uneven path, allowing the occupant and caregiver to walk side by side. The people depicted in the video gave written consent to publish as outlined in the PLOS consent form.
(MP4)
