# Supplementary material for: A slanted castor wheel enables pushing manual wheelchairs from the side to improve social interaction
Source: PLoS One. 2024 Sep 12;19(9):e0307759. doi: 10.1371/journal.pone.0307759 (PMC11392412; doi:10.1371/journal.pone.0307759)
Supplement: S1 Appendix — (PDF) [file pone.0307759.s001.pdf]

S1 Appendix. Description of kinematics and tire forces

List of assumptions

In the system definition and in describing the cosine approximation of the simplified castor wheel, a number of assumptions are made about the wheelchair and environment. All assumptions are summarized here:

Assumption 1 *The weight of the castor fork and wheel is negligible compared to the weight of the vehicle.*

Assumption 2 *The wheels are modelled as flat discs and have a single contact point.*

Assumption 3 *The wheels have no turning resistance, so the aligning moment transmitted at contact in direction $\hat{\boldsymbol{n}}$ is zero. The justification for its omission is given in the "Rolling contact forces of a castor wheel" subsection.*

Assumption 4 *The wheels have no overturning moment M_l as the wheel is modelled as a flat disc, so no moment is transmitted at contact in direction $\hat{\boldsymbol{l}}$.*

Assumption 5 *Rolling resistance has no viscous components. It can be modelled as a longitudinal tire force F_l with a fixed rolling resistance coefficient as described by Sauret et al. [1], and a rolling resistance moment M_t [2].*

Assumption 6 *The wheels do not slip in the longitudinal or lateral direction. The effects of lateral slip (e.g. slip angle) are discussed in the "Rolling contact forces of a castor wheel" subsection.*

Assumption 7 *The bearings of the wheel axles and swivel axis have no friction.*

Assumption 8 *The ground plane is flat and perpendicular to gravity.*

Assumption 9 *The small height change induced by varying the swivel angle under a cant or rake angle does not significantly alter the vehicle's pitch angle. As a result, the vehicle is always parallel to the ground plane and can only rotate in the yaw direction with angle ζ_z.*

Assumption 10 *The vehicle is in a steady-state movement with constant forward and angular velocity.*

The following additional assumptions were made to derive the simplified Eq 13. The aim of these additional simplifications was to provide a short equation that could broadly capture the effects of the modification. We emphasize that these are large simplifications of the kinematics and may not be valid for any castor wheel or vehicle.

Assumption 11 *The small-angle approximation can be applied to φ_x and φ_y. The camber angle γ is bounded by the previous two angles and can be assumed to be small as well.*

Assumption 12 *The contact point's nonlinear kinematics as a function of the rake and cant angle can be ignored. This greatly simplifies the moment arm compared to what it was in a regular castor wheel.*

Assumption 13 *The roll resistance moment M_t only causes a small moment in the direction of the swivel axis, which is negligible.*

Definitions of rotations and triads

The vehicle, castor fork, and castor wheel can rotate with respect to the fixed world. To keep track of their rotation, each body is assigned a triad, denoted with calligraphic characters as listed in Fig 1. Rotations between triads are defined using rotation matrices $\mathbf{R}$, following the definition that

$$^{\mathcal{N}}\boldsymbol{r} = {^{\mathcal{N}}\mathbf{R}_{\mathcal{A}}}\,{^{\mathcal{A}}\boldsymbol{r}}, \tag{S1}$$

where $\boldsymbol{r}$ is an arbitrary vector.

Not all rotations between triads can be chosen arbitrarily, as joints and a closed kinematic loop constrain the system. The fixed-world triad $\mathcal{N}$ is shown at the start and end of the rotations in Fig 1. The closed loop is opened by solving the angles ϵ, γ, and β as functions of the other angles.

It is assumed that the vehicle's orientation is always aligned with the ground plane, meaning it can only rotate around the inertial z-axis with ζ_z.

$$^{\mathcal{B}}\mathbf{R}_{\mathcal{N}} = \begin{pmatrix} c_{\zeta_z} & s_{\zeta_z} & 0 \\ -s_{\zeta_z} & c_{\zeta_z} & 0 \\ 0 & 0 & 1 \end{pmatrix} \tag{S2}$$

The castor fork can swivel with respect to the vehicle along the swivel axis. The orientation of this swivel axis is parameterized using the rake angle φ_x and the cant angle φ_y. The swivel angle δ is used to encode the swivel rotation of the fork with respect to the vehicle.

$$^{\mathcal{D}}\mathbf{R}_{\mathcal{B}} = \begin{pmatrix} 1 & 0 & 0 \\ 0 & c_{\varphi_x} & s_{\varphi_x} \\ 0 & -s_{\varphi_x} & c_{\varphi_x} \end{pmatrix} \begin{pmatrix} c_{\varphi_y} & 0 & -s_{\varphi_y} \\ 0 & 1 & 0 \\ s_{\varphi_y} & 0 & c_{\varphi_y} \end{pmatrix} \tag{S3}$$

$$^{\mathcal{R}}\mathbf{R}_{\mathcal{D}} = \begin{pmatrix} c_{\delta} & s_{\delta} & 0 \\ -s_{\delta} & c_{\delta} & 0 \\ 0 & 0 & 1 \end{pmatrix} \tag{S4}$$

The wheel bank angle σ is the angle between the wheel spin axis and the $\hat{\boldsymbol{r}}_2$ vector from triad $\mathcal{F}$. In traditional castor wheels, the swivel axis lies in the wheel's plane, corresponding to a σ of zero.

$$^{\mathcal{F}}\mathbf{R}_{\mathcal{R}} = \begin{pmatrix} 1 & 0 & 0 \\ 0 & c_{\sigma} & s_{\sigma} \\ 0 & -s_{\sigma} & c_{\sigma} \end{pmatrix} \tag{S5}$$

Lastly, the angle θ encodes the wheel's rotation with respect to the castor fork.

$$^{\mathcal{W}}\mathbf{R}_{\mathcal{R}} = \begin{pmatrix} c_{\theta} & 0 & -s_{\theta} \\ 0 & 1 & 0 \\ s_{\theta} & 0 & c_{\theta} \end{pmatrix} \tag{S6}$$

The rotations for triads $\mathcal{A}$ and $\mathcal{C}$ could be defined using ϵ, γ, and β, but these angles can not be chosen independently of the previously presented rotations. Instead, the rotation matrix $^{\mathcal{C}}\mathbf{R}_{\mathcal{F}}$ from the castor fork to the instantaneous contact point can be calculated by finding the components of $\mathcal{C}$ expressed in $\mathcal{F}$.

$$^{\mathcal{C}}\mathbf{R}_{\mathcal{F}} = \begin{pmatrix} {^{\mathcal{F}}\hat{\boldsymbol{l}}^{\mathrm{T}}} \\ {^{\mathcal{F}}\hat{\boldsymbol{t}}^{\mathrm{T}}} \\ -{^{\mathcal{F}}\hat{\boldsymbol{n}}^{\mathrm{T}}} \end{pmatrix} \tag{S7}$$

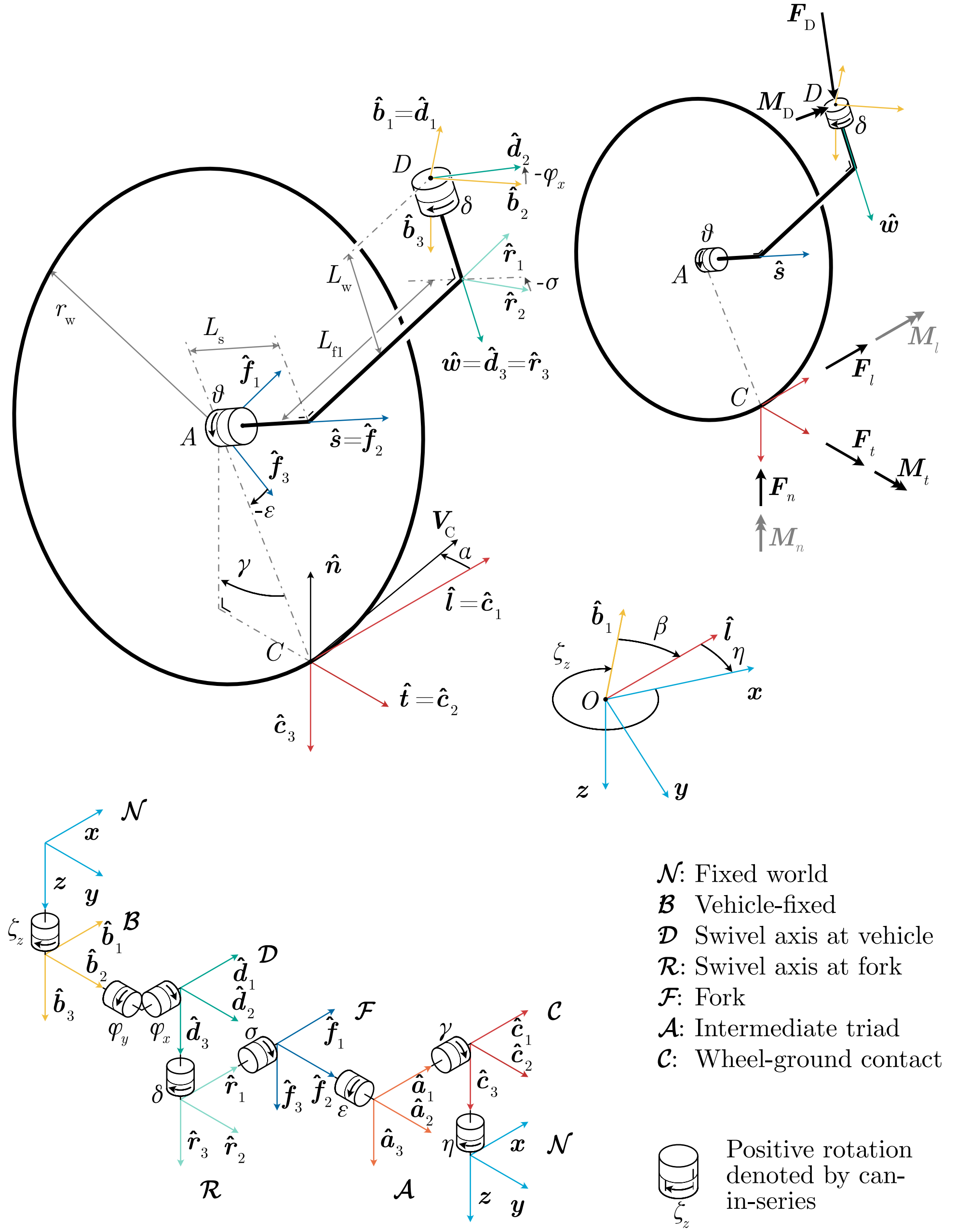

Fig 1. Full kinematic and free body diagram of a modified castor wheel, including the wheel slip angle α. *Top left*: Kinematic diagram. Point D is the connection to the vehicle, point A is the connection between the castor fork and wheel, and C is the geometric contact point. *Top right*: Free body diagram of the castor wheel. The force and moment on point D act on the vehicle, and the contact forces and moments at point C act on the ground. Moments that are ignored are colored gray. *Bottom*: A cans-in-series [3] representation of the orientations of triads with respect to each other. The arrow on each can (cylinder) indicates the direction of a positive rotation when the triad on the other side is fixed. In cases where multiple consecutive rotations are performed between triads, the shown cans-in-series represent the order of the Euler angles yaw, pitch, and roll. Because the fixed world coordinate system $\mathcal{N}$ appears on both ends of the kinematic chain, not all angles can be chosen independently.

The normal vector $\hat{\boldsymbol{n}}$ and wheel spin vector $\hat{\boldsymbol{s}}$ are defined by the inertial coordinate system $\mathcal{N}$ and castor fork triad $\mathcal{F}$ respectively. The rotation matrices described above can be used to express the unit vectors in the $\mathcal{F}$ triad.

$$ {}^{\mathcal{N}}\hat{\boldsymbol{n}} = \begin{pmatrix} 0 \\ 0 \\ -1 \end{pmatrix} \tag{S8} $$

$$ {}^{\mathcal{F}}\hat{\boldsymbol{s}} = \begin{pmatrix} 0 \\ 1 \\ 0 \end{pmatrix} \tag{S9} $$

The longitudinal contact direction of the wheel $\hat{\boldsymbol{l}}$ must be perpendicular to the wheel spin axis $\hat{\boldsymbol{s}}$ and the ground normal vector $\hat{\boldsymbol{n}}$. The product is divided by the norm as $\hat{\boldsymbol{n}}$ and $\hat{\boldsymbol{s}}$ are not perpendicular.

$$ \hat{\boldsymbol{l}} = \frac{-\hat{\boldsymbol{s}} \times \hat{\boldsymbol{n}}}{||\hat{\boldsymbol{s}} \times \hat{\boldsymbol{n}}||} \tag{S10} $$

The lateral direction $\hat{\boldsymbol{t}}$ can be determined with a cross product, without dividing by the norm, as both unit vectors are orthogonal by definition.

$$ \hat{\boldsymbol{t}} = \hat{\boldsymbol{l}} \times \hat{\boldsymbol{n}} \tag{S11} $$

Using these unit vectors, Eq S7 can be solved. With this rotation found, all relevant rotations to find the equations of motion of the castor wheel have been defined.

Definitions of positions

Distances between points are defined using position vectors in the form of ${}^{\mathcal{N}}\boldsymbol{r}_A$, where $\mathcal{N}$ indicates in which triad the position vector is expressed, and A is the location of the point with respect of the origin O. For example, a relative position vector ${}^{\mathcal{N}}\boldsymbol{r}_{A/B}$ is the vector pointing from point B to point A.

The vehicle's position is described using its origin, point B. The x and y coordinates of the vehicle origin with respect to the origin O are described by generalized coordinates x_B, y_B, and z_B. Recall that the positive z axis of the inertial coordinate system $\mathcal{N}$ points into the ground.

$$ {}^{\mathcal{N}}\boldsymbol{r}_{\mathrm{B/O}} = {}^{\mathcal{N}}\boldsymbol{r}_{\mathrm{B}} = \begin{pmatrix} x_B \\ y_B \\ z_B \end{pmatrix} \tag{S12} $$

Point D is the connection between the wheelchair and the castor wheel. Due to the small angle approximation for the pitch angle of the wheelchair, the Z coordinate of this point is free with respect to point B, and governed by generalized coordinate z_D.

$$ {}^{\mathcal{N}}\boldsymbol{r}_D = \begin{pmatrix} x_B \\ y_B \\ z_B \end{pmatrix} + {}^{\mathcal{N}}\mathbf{R}_{\mathcal{B}} \begin{pmatrix} a_{f1} \\ b_{f1} \\ z_D \end{pmatrix} \tag{S13} $$

Point A is the connection between the castor fork and the castor wheel, and it can be found using the length of the swivel axis, L_w, and the castor trail lengths L_{f1} and L_s. Point F is an intermediate point on the castor fork.

$$ {}^{\mathcal{N}}\boldsymbol{r}_F = {}^{\mathcal{N}}\boldsymbol{r}_D + {}^{\mathcal{N}}\mathbf{R}_{\mathcal{D}} {}^{\mathcal{D}}\boldsymbol{r}_{F/D} = {}^{\mathcal{N}}\boldsymbol{r}_D + {}^{\mathcal{N}}\mathbf{R}_{\mathcal{D}} \begin{pmatrix} 0 \\ 0 \\ L_w \end{pmatrix} \tag{S14} $$

$$ {}^{\mathcal{N}}\boldsymbol{r}_A = {}^{\mathcal{N}}\boldsymbol{r}_F + {}^{\mathcal{N}}\mathbf{R}_{\mathcal{F}}\,{}^{\mathcal{F}}\boldsymbol{r}_{A/F} = {}^{\mathcal{N}}\boldsymbol{r}_D + {}^{\mathcal{N}}\mathbf{R}_{\mathcal{F}} \begin{pmatrix} -L_{f1} \\ L_s \\ 0 \end{pmatrix} \tag{S15} $$

The relative position of point C with respect to point A can be found by taking the cross product of $\hat{\boldsymbol{l}}$ and $\hat{\boldsymbol{s}}$. Since $\hat{\boldsymbol{l}}$ and $\hat{\boldsymbol{s}}$ are perpendicular unit vectors, it is not needed to divide by the norm, resulting in:

$$ {}^{\mathcal{N}}\boldsymbol{r}_C = {}^{\mathcal{N}}\boldsymbol{r}_A + ({}^{\mathcal{N}}\hat{\boldsymbol{l}} \times^{\mathcal{N}} \hat{\boldsymbol{s}}) \cdot r_w, \tag{S16} $$

where r_w is the radius of the wheel. This formulation describes the location of point C with respect to the origin. For calculations of the castor wheel it is also useful to know the location of point C with respect to the vehicle connection point D. This is equal to

$$ {}^{\mathcal{N}}\boldsymbol{r}_{C/D} = {}^{\mathcal{N}}\mathbf{R}_{\mathcal{D}} \begin{pmatrix} 0 \\ 0 \\ L_w \end{pmatrix} + {}^{\mathcal{N}}\mathbf{R}_{\mathcal{F}} \begin{pmatrix} -L_{f1} \\ L_s \\ 0 \end{pmatrix} + ({}^{\mathcal{N}}\hat{\boldsymbol{l}} \times^{\mathcal{N}} \hat{\boldsymbol{s}}) \cdot r_w. \tag{S17} $$

Constrained angles of the closed kinematic loop

Since the kinematic chain shown in Fig 1 is closed, the camber angle γ, fork pitch angle ϵ, and heading angle β cannot be determined independently. Instead, they are derived using the orientation of known unit vectors. The four-quadrant inverse tangent can be used to calculate the angles unambiguously:

$$ \epsilon = \operatorname{atan2}(\hat{\boldsymbol{l}} \cdot \hat{\boldsymbol{f}}_1, \hat{\boldsymbol{l}} \cdot \hat{\boldsymbol{f}}_3), \tag{S18} $$

$$ \gamma = -\operatorname{atan2}(\hat{\boldsymbol{s}} \cdot \hat{\boldsymbol{n}}, \hat{\boldsymbol{s}} \cdot \hat{\boldsymbol{t}}), \tag{S19} $$

$$ \beta = \operatorname{atan2}(\hat{\boldsymbol{l}} \cdot \boldsymbol{b}_2, \hat{\boldsymbol{l}} \cdot \boldsymbol{b}_1). \tag{S20} $$

These angles are uniquely defined in the range $[-\pi, \pi]$ for all cases except in gimbal lock, which occurs in the unlikely situation where the wheel and ground planes coincide.

In a simple castor wheel with a small cant angle where the lateral trail, rake angle, and wheel bank angle are zero, these angles simplify to

$$ \epsilon \approx -\varphi_x \sin(\delta), \tag{S21} $$

$$ \gamma \approx \varphi_x \cos(\delta), \tag{S22} $$

$$ \beta \approx \delta. \tag{S23} $$

Rolling contact forces of a castor wheel

Rolling contact results in contact forces and moment in all principal directions of the contact triad $\mathcal{C}$ [2]. In rolling contact, there often is a small difference between the heading of the wheel $\hat{\boldsymbol{l}}$ and the instantaneous direction of movement of the wheel $\boldsymbol{V}_C$. The angle between the heading and velocity of the wheel is called the *slip angle* α. This lateral slip results in an increase in the lateral force F_t. The *cornering stiffness* of a wheel quantifies how much lateral force the wheel generates at a given slip angle and normal force F_n. Even in transient conditions, little force is required due to the small mass and inertia of a castor wheel [4]. However, the larger lateral force may increase the slip angle for a castor wheel with a nonzero cant angle. The slip angle was assumed to be zero in all calculations in this manuscript.

The *camber thrust* is the component of the lateral force that is generated by lateral compression of the wheel due to the angle between the ground plane and the wheel,

called the camber angle γ, The contribution to the force can be approximated as the normal force F_n multiplied by the tangent of the camber angle [5–8]. This approximation is also called the tangent rule and is based on the idea that a wheel could be modeled as a "brush" of linear springs that reach out from the wheel's axle. Because the camber thrust is generated by the compression of these springs in the plane of the wheel, the corresponding force should be in this plane as well [7]. For small camber angles, the tangent of γ is equal to γ. When taking both camber thrust and cornering stiffness into account, the lateral force is equal to:

$$F_t = F_n \left(\gamma + C_{F\alpha}\alpha\right). \tag{S24}$$

The lateral force F_t consists of the camber thrust, which was assumed to be a function of only the camber angle γ in Eq S24, and the cornering force of the wheel parameterized by the slip angle α and cornering stiffness. The reaction force is within the plane of the wheel, while the slip angle is zero due to the camber thrust. An increase in the cant angle also increases the camber angle between the wheel and the ground. The camber thrust increases proportionally, and due to this match between the cant angle and camber thrust, the slip angle remains negligible. However, the slip angle increases when the wheel bank angle is nonzero. Based on this observation, we conclude that the wheel bank angle should be kept near zero and that the cant angle is more suitable for generating lateral tire force.

The *aligning torque* M_z as a result of stiffness or camber angle is very small when compared to the other effects; the coefficients reported by Dressel [7] are lower than 0.005 and 0.0002 normalized torque per degree respectively for almost all measured bicycle wheels. To the authors' knowledge, no similar experiments have been performed using wheelchair castor wheels. By comparison, the cornering stiffness for F_t tends to be around 0.2 normalized force per degree slip. Frank and Abel [9] report a static turning resistance of less than 0.01 Nm per N of vertical load for any tested floor type. Therefore, the contribution of M_z is safely ignored.

Lastly, we calculate the magnitude of the rolling resistance moment M_t using a fixed rolling resistance coefficient. The rolling resistance coefficient f_r is often determined by dividing the force required to pull the wheel by the normal force, and was performed before for front and rear manual wheelchair wheels [1]. We assume the rolling resistance coefficient does not depend on the camber angle γ. Since there is no friction in the wheel spin bearing, the rolling resistance moment must be opposite to the moment generated by the longitudinal tire force. The longitudinal force and rolling resistance moment are then equal to

$$F_l = -F_n\, f_r, \tag{S25}$$

$$M_t = -F_l\, r_w, \tag{S26}$$

where f_r is the rolling resistance coefficient, and r_w is the radius of the castor wheel.

With all contact forces and moments determined, the moment on the connection with the vehicle can be determined:

$$\boldsymbol{M}_D = -\boldsymbol{r}_{C/D} \times \boldsymbol{F}_C - \boldsymbol{M}_t. \tag{S27}$$

References

1. Sauret C, Bascou J, de Saint Remy N, Pillet H, Vaslin P, Lavaste F. Assessment of field rolling resistance of manual wheelchairs. Journal of Rehabilitation Research and Development. 2012;49:63–74. doi:10.1682/JRRD.2011.03.0050.

2. Pacejka HB, Besselink I. Tire and Vehicle Dynamics. 3rd ed. Elsevier; 2012.

3. Schwab AL, Meijaard JP. How to Draw Euler Angles and Utilize Euler Parameters. vol. Volume 2: 30th Annual Mechanisms and Robotics Conference, Parts A and B of International Design Engineering Technical Conferences and Computers and Information in Engineering Conference; 2006. p. 259–265. Available from: `https://doi.org/10.1115/DETC2006-99307`.

4. Doria A, Taraborrelli L, Jomaa T, Peijs T, Potter M, Advani S, et al. Identification of the Mechanical Properties of Tires for Wheelchair Simulation. The Open Mechanical Engineering Journal. 2016;10(1):183–200. doi:10.2174/1874155x01610010183.

5. Sakai H, Kanaya O, Iijima H. Effect of Main Factors on Dynamic Properties of Motorcycle Tires. In: SAE Technical Paper Series. SAE International; 1979.Available from: `https://doi.org/10.4271/790259`.

6. Pacejka HB, Sharp RS. Shear Force Development by Pneumatic Tyres in Steady State Conditions: A Review of Modelling Aspects. Vehicle System Dynamics. 1991;20(3-4):121–175. doi:10.1080/00423119108968983.

7. Dressel AE. Measuring and Modeling the Mechanical Properties of Bicycle Tires. University of Wisconsin-Milwaukee; 2013. Available from: `https://dc.uwm.edu/etd/386/`.

8. Sharp RS. On the Stability and Control of the Bicycle. Applied Mechanics Reviews. 2008;61(6). doi:10.1115/1.2983014.

9. Frank TG, Abel EW. Measurement of the turning, rolling and obstacle resistance of wheelchair castor wheels. Journal of Biomedical Engineering. 1989;11(6):462–466. doi:10.1016/0141-5425(89)90040-x.
